# Analysis of the Genetic Diversity in Tea Plant Germplasm in Fujian Province Based on Restriction Site-Associated DNA Sequencing

**DOI:** 10.3390/plants13010100

**Published:** 2023-12-28

**Authors:** Lele Jiang, Siyi Xie, Chengzhe Zhou, Caiyun Tian, Chen Zhu, Xiaomei You, Changsong Chen, Zhongxiong Lai, Yuqiong Guo

**Affiliations:** 1 College of Horticulture, Fujian Agriculture and Forestry University, Fuzhou 350002, China; lelejiang0625@foxmail.com (L.J.); chengzhechou@foxmail.com (C.Z.); cytian1997@foxmail.com (C.T.); laizx01@163.com (Z.L.); 2 Key Laboratory of Tea Science of Ministry of Education, Hunan Agricultural University, Changsha 410128, China; siyixie979@foxmail.com; 3 Institute of Horticultural Biotechnology, Fujian Agriculture and Forestry University, Fuzhou 350002, China; 4 Guangdong Provincial Key Laboratory of Applied Botany & Key Laboratory of South China Agricultural Plant Molecular Analysis and Genetic Improvement, South China Botanical Garden, Chinese Academy of Sciences, No. 723 Xingke Road, Tianhe District, Guangzhou 510650, China; zhuchen19921118@foxmail.com; 5 Tea Research Institute, Fujian Academy of Agricultural Sciences, No. 104 Pudang Road, Xindian Town, Jin’an District, Fuzhou 350012, China; yxm0593@163.com (X.Y.); ccs6536597@163.com (C.C.); 6 Anxi College of Tea Science (College of Digital Economy), Fujian Agriculture and Forestry University, Quanzhou 362400, China

**Keywords:** *Camellia sinensis*, genetic diversity, selection pressure, genome-wide association analysis

## Abstract

Fujian province, an important tea-producing area in China, has abundant tea cultivars. To investigate the genetic relationships of tea plant cultivars in Fujian province and the characteristics of the tea plant varieties, a total of 70 tea cultivars from Fujian and other 12 provinces in China were subjected to restriction site-associated DNA sequencing (RAD-seq). A total of 60,258,975 single nucleotide polymorphism (SNP) sites were obtained. These 70 tea plant cultivars were divided into three groups based on analyzing the phylogenetic tree, principal component, and population structure. Selection pressure analysis indicated that nucleotide diversity was high in Southern China and genetically distinct from cultivars of Fujian tea plant cultivars, according to selection pressure analysis. The selected genes have significant enrichment in pathways associated with metabolism, photosynthesis, and respiration. There were ten characteristic volatiles screened by gas chromatography–mass spectrometry (GC–MS) coupled with multivariate statistical methods, among which the differences in the contents of methyl salicylate, 3-carene, cis-3-hexen-1-ol, (*E*)-4-hexen-1-ol, and 3-methylbutyraldehyde can be used as reference indicators of the geographical distribution of tea plants. Furthermore, a metabolome genome-wide association study (mGWAS) revealed that 438 candidate genes were related to the aroma metabolic pathway. Further analysis showed that 31 genes of all the selected genes were screened and revealed the reasons for the genetic differences in aroma among tea plant cultivars in Fujian and Southern China. These results reveal the genetic diversity in the Fujian tea plants as well as a theoretical basis for the conservation, development, and utilization of the Fujian highly aromatic tea plant cultivars.

## 1. Introduction

The tea plant (*Camellia sinensis* (L.) O. Kuntze), an evergreen woody plant that originated in Southwest China, has been cultivated for approximately 3000 years and is now widely cultivated in more than 50 countries and regions around the world [1]. The majority of cultivated tea plants belong to the genus *Camellia* L., section *Thea* (L.) Dyer, in the family Theaceae and are categorized as one of two main varieties: *C. sinensis* var. *sinensis* (CSS) and *C. sinensis* var. *assamica* (Masters) Chang (CSA) [2]. The tea plants in China are mainly grown in Southwest China, Southern China, south of the Yangtze River, and north of the Yangtze River regions [3]. Fujian province is abundant in tea plant cultivars and tea varieties [4]. It is the birthplace of oolong tea, black tea, and white tea among the six tea categories, and the aroma for a relatively high proportion in the quality evaluation, especially in oolong tea [5,6]. Subsequent to its initial domestication, tea was further bred and cultivated to enhance certain organoleptic traits, primarily taste and aroma, as well as biotic and abiotic stress resistance properties [7]. Among these, aroma is an important indicator of the flavor quality of tea, determined by the genetic characteristics of tea plant cultivars. The components and contents of aroma are influenced by gene sequence, gene expression, and other factors, with gene sequence variation in different species being the constitutive factor that directly influences aroma [8,9,10]. The rapid and precise identification of genes that provide a favorable expression of traits is a high priority, and while genes related to tea plant type and leaf phenotype have been identified by researchers [11,12,13], fewer studies have been conducted to analyze the causes of aroma differences in different tea plant cultivars from the perspective of tea plant genetic diversity.

With the rapid development of high-throughput sequencing technology, reduced-representation genome sequencing (RRGS) has been widely used as an efficient marker development technology [14,15]. Amongst a significant amount of technology, thousands of SNP markers have been obtained using restriction site-associated DNA sequencing (RAD-seq) [16]. Additionally, with the development of mass spectrometry (MS) platforms and sequencing technology, metabolome-based genome-wide association studies (mGWAS) have successfully been applied to exploit natural genotypic variation and identify metabolic quantitative trait loci (mQTL) responsible for metabolomic variation in plants [17,18,19,20,21]. Based on RRGS data, the phylogenetic relationships, genetic diversity, and geographical distribution of tea plants have been revealed [11,22,23,24]. Analyses of genetic diversity and the identification of genes associated with excellent traits play an essential role in the breeding of superior varieties. Recently, based on mGWAS, the metabolic diversity of tea plants was revealed and the biosynthesis of tea plant flavonoids was further updated [17]. Genome-wide association analysis was conducted on 11 leaf-related traits of ancient tea plant populations in Yunnan and Guizhou, and four candidate genes that may be involved in regulating plant size, leaf color, and other related traits were screened [11]. Nevertheless, metabolite diversity among different tea plant cultivars and the identification of related genes remain to be explored.

In this study, the genetic relationships of 70 tea plant cultivars from Fujian and other 12 provinces in China were analyzed by RAD-seq to reveal the origin and evolutionary spread of tea plants in Fujian province. In addition, we also examined the volatiles of 70 tea plant cultivars, conducted mGWAS analysis of tea plant characteristic aroma traits based on SNP loci, and combined this with selection pressure analysis to investigate the causes of genetic differences in the aroma of different tea plant cultivars and accurate positioning key genes related to the aroma forming in the tea plant, which will provide scientific reference for the selection and breeding of tea plant cultivars with highly aromatic aromas [25].

## 2. Result

### 2.1. Overview of Sequencing Quality of 70 Tea Plant Cultivars

**Figure 1:**
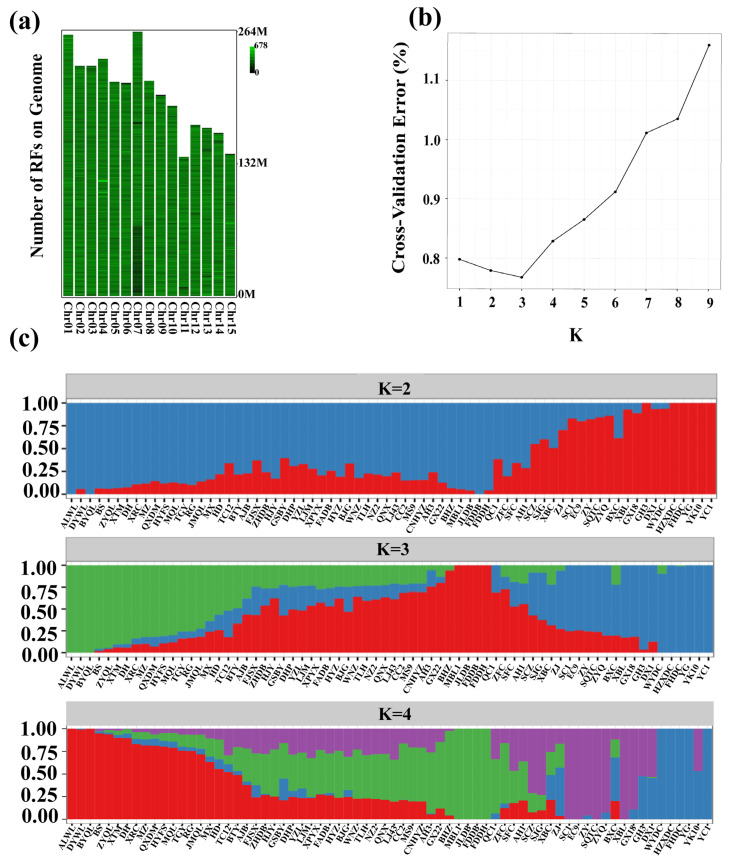
Population structure of 70 tea plant cultivars. (**a**) Effective-RF chromosome distribution map. (**b**) Cross-validation of K values. (**c**) Stacked diagram of tea plant community structure at K = 2, K = 3, and K = 4.

Seventy tea cultivars were used for the analysis of this study (Supplemental Table S1). The “Tieguanyin” genome (PRJCA003090) was selected as the reference genome by comparison of published genomic data [26]. A total of 1,686,990 restriction enzyme fragments were obtained by e-enzymatic digestion, and 1,135,399 effective restriction enzyme fragments were obtained. The effective restriction enzyme fragments were uniformly distributed in the genome, and the enzyme cutting exhibited a positive effect by using restriction endonuclease EcoR I, which can be used for library construction and sequencing (Figure 1a). After sequencing, a total of 3.02 Gb reads of raw sequence data were obtained from 70 tea plant cultivars and 2.97 Gb of clean reads were valid after filtering (Supplemental Table S2). The average proportion of sequenced Q30 was 92%, the average of GC was 40%, and the sequence length of each sample was 150. The filtered high-quality reads were compared with the reference genome, and the comparison rate averaged 97.55%, which indicated that the distribution of base content was normal, and the quality of sequencing was high enough to meet the needs of subsequent analysis (Supplemental Table S3).

### 2.2. SNP Variation and Genetic Relationships Analysis of 70 Tea Plant Cultivars

A total of 60,258,975 SNP loci were detected in the 70 tea plant cultivars, of which 59,666,249 SNP loci were distributed on 15 chromosomes (Supplemental Table S4). The conversion-to-subversion ratio (Ts/Tv) was 1.8, with a higher conversion rate of 20.33% for C/T (12,724,288) (Supplemental Figure S1a). In addition, the number of insertion and deletion variants were detected as 1,009,010 and 1,528,058, respectively, with lengths mainly ranging from 1 to 8 bp and from 1 to 2 bp (Supplemental Figure S1b).

**Figure 2:**
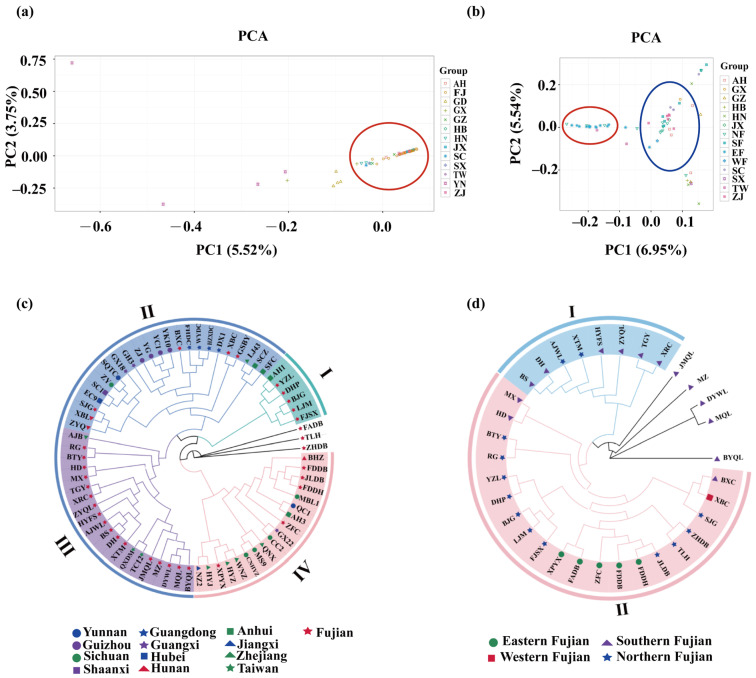
(**a**) Principal component analysis (PCA) of 70 tea plant cultivars; (**b**) The PCA of the overlapping segment in figure (**a**); (**c**) Phylogenetic tree of 70 tea plant cultivars; (**d**) Phylogenetic tree of tea plant cultivars in Fujian province. AH: Anhui; FJ: Fujian; GD: Guangdong; GX: Guangxi; GZ: Guizhou; HB: Hubei; HN: Hunan; JX: Jiangxi; SC: Sichuan; SX: Shaanxi; TW: Taiwan; YN: Yunnan; ZJ: Zhejiang; NF: Northern Fujian; EF: Eastern Fujian; SF: Southern Fujian; WF: Western Fujian.

To illustrate the genetic relationship of 70 tea populations, population structure, principal component analysis (PCA), and a phylogenetic tree were constructed based on the SNPs. The values of cross-validation (CV) error were calculated for each K to select an optimal number of populations. The results show that the CV value reached its lowest value when K = 3, which indicated that the optimal number of populations should be three (Figure 1b,c). The results of the principal component analysis showed (Figure 2a) that some tea plants in Yunnan, Guangdong, and Guangxi provinces were distributed far away from other local tea plants. However, there was a severe overlap between Fujian province tea plants and the rest of the tea plant cultivars from other provinces. Therefore, further analysis of the overlapping areas (Figure 2b) revealed that group 1 was mainly composed of tea plant cultivars from Southern Fujian, Northern Fujian, and Taiwan, whereas group 2 was mainly composed of tea plant cultivars from Eastern Fujian, Northern Fujian, Zhejiang, Anhui, and Sichuan. The genetic diversity of 70 tea plant cultivars was also analyzed by conducting a phylogenetic tree. Based on the SNPs, 70 tea plant cultivars were classified into three subgroups (Figure 2c), which were in accordance with the results of the population structure and PCA. To further analyze the genetic relationship of tea plant cultivars in Fujian province, 34 tea plant cultivars in Fujian province were individually constructed into a phylogenetic tree. The results show (Figure 2d) that all the 34 tea plant cultivars were divided into two groups. Most of the tea plant cultivars from Southern Fujian and two from Northern Fujian were clustered into group I. All the tea plant cultivars from Eastern Fujian and most of the tea plant cultivars from Northern Fujian were clustered into one subgroup in group II. Meanwhile, the XBC from Western Fujian and BXC from Southern Fujian are closely related. The remaining tea plant cultivars from Southern Fujian are independent of other cultivars. All the above results are consistent with the results of the phylogenetic tree constructed from 70 tea plant cultivars.

### 2.3. Selective Pressure Analysis

Based on the filtered SNPs, nucleotide diversity (π) and genetic differentiation index (Fst) were calculated for Fujian, Eastern China excluding Fujian (EC-F), Southern China, Central China, and Western China (Supplemental Table S1) [27]. The π-value and Fst were both higher in Fujian and Southern China compared to the other three regions, indicating the greatest differentiation between them (Supplemental Figure S2). To explore the differences between tea plant cultivars in Fujian and other regions, the pairwise comparison of tea plant cultivars in Fujian and other regions was conducted for selection pressure analysis. There, 202, 158, 295, and 180 genes were screened in the selected regions from EC-F and Fujian (ECF), Southern China and Fujian (SCF), Central China and Fujian (CCF), and Western China and Fujian (WCF), respectively (Supplemental Figures S4 and S5).

To further explore the functions of the selected genes, these genes were annotated based on GO and KEGG databases. The GO enrichment analysis results show that the selected genes in the four subgroups were mainly enriched in the pathways related to respiration and photosynthesis and had the highest enrichment associated with cellular components (Supplemental Figure S6). KEGG enrichment analysis of the selected genes showed (Supplemental Figure S7) that the top 20 KEGG enrichment pathways were involved in phytohormone signaling, plant–pathogen interactions, phenylpropane biosynthesis, fatty acid biosynthesis, respiration, photosynthesis, etc. There were a large number of functionally enriched genes in respiration, photosynthesis, and other metabolism-related pathways in each subgroup, and the metabolic pathway had the largest number of enriched genes, which was consistent with the results of GO enrichment analysis. The selected genes related to the four subgroups of metabolic pathways were compared and analyzed together with the GO enrichment analysis results, and three genes (*Peroxidase 4-like*, *ATPA*, and *COX2*) were found to be selected in both ECF and SCF, among which *ATPA* and *COX2* were consistent with the GO annotation results in both regions. Two genes, *ND1* and *ND2*, were selected in both SCF and WCF. Three genes (*GLU3*, *HEXO3*, and *SAMDC*) were selected in CCF and ECF, and one gene (*PCAS-1*) was selected in CCF and WCF.

### 2.4. Overall Profile of Volatiles in 70 Tea Plant Cultivars

**Figure 3:**
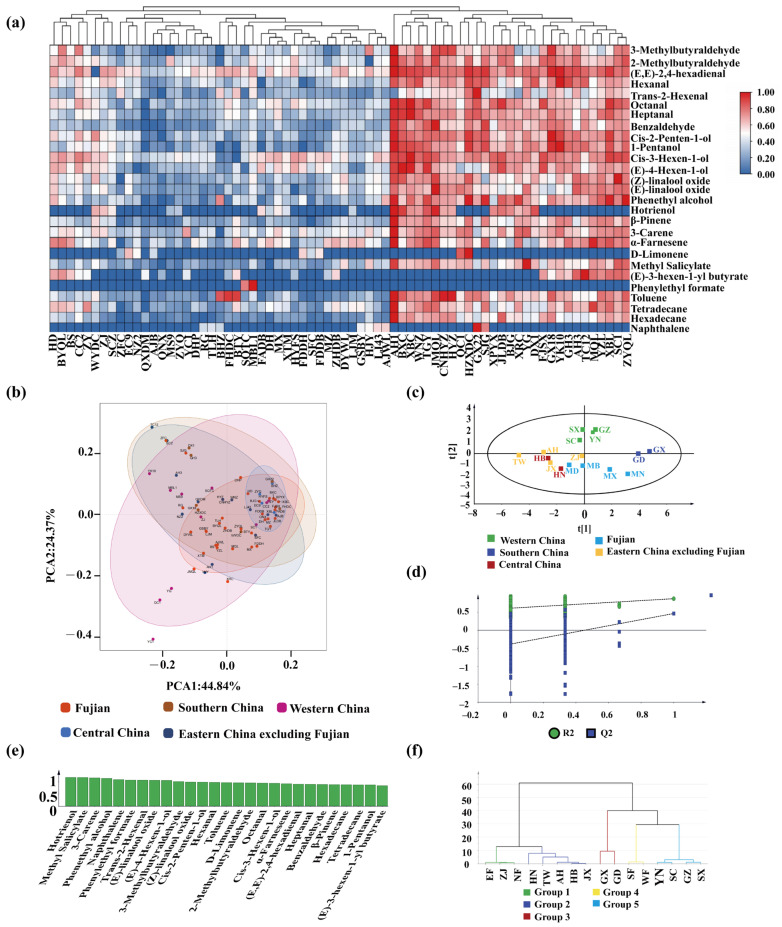
PCA of volatile and orthogonal projections to latent structures discriminant analysis (OPLS-DA) of volatiles in fresh tea leaves. (**a**) A hierarchical clustering analysis (HCA) heatmap of volatiles of 70 tea plants; (**b**) PCA of volatile in fresh tea leaves; (**c**) OPLS-DA score plot; (**d**) Permutation test plot; (**e**) The variable importance in the project (VIP) of 27 volatiles; (**f**) HCA plot (EF: Eastern Fujian; ZJ: Zhejiang; NF: Northern Fujian; HN: Hunan; TW: Taiwan; AH: Anhui; HB: Hubei; JX: Jiangxi; GX: Guangxi; GD: Guangdong; SF: Southern Fujian; WF: Western Fujian; YN: Yunnan; SC: Sichuan; GZ: Guangzhou; SX: Shaanxi).

A total of 27 major volatiles were detected in the 70 tea leaves by GC–MS and classified based on the composition of the volatiles, including eight alcohols, eight aldehydes, eight hydrocarbons, and three lipids (Supplemental Table S5). The 20 volatiles were common to 70 tea plants, and the total volatile contents ranged from 688.26 to 125,924.90 μg/kg. To explore the differences in volatiles of tea plant cultivars under different geographical origins, the proportions of volatile content of each category were analyzed and it was found that the volatiles in all regions were mainly alcohols and carbon compounds (Supplemental Figure S8). A hierarchical clustering analysis (HCA) heatmap was conducted based on the volatile content of all tea plant cultivars, showing that 70 tea plant cultivars were clustered into two groups (Figure 3a). The overall volatile contents in the first category were lower than that in the second category, which mainly contains tea plant cultivars from Fujian, Sichuan, and Zhejiang, with the highest content of *cis*-3-hexen-1-ol and (*E*,*E*)-2,4-hexadienal. Tea plant cultivars from ten provinces (Anhui, Fujian, Zhejiang, Sichuan, Guizhou, Guangdong, Guangxi, Yunnan, Taiwan, and Shaanxi) were clustered into the second category, with a higher content of 3-carene and (*E*)-4-hexen-1-ol.

### 2.5. Analysis of Characteristic Volatile in 70 Tea Plant Cultivars

The contributions of volatiles to tea aroma cannot completely depend on the amount of each volatile; therefore, we used odor activity value (OAV ≥ 1) to calculate the significance of the characteristic volatiles in 70 tea cultivars (Supplemental Table S6). The results show that a total of 15 volatiles revealed OAVs ≥ 1 in 70 tea cultivars, including seven aldehydes, four alcohols, three hydrocarbons, and one ester. Most of the aldehydes presented fresh and fruity aromas, among them, 3-methylbutyraldehyde, 2-methylbutyraldehyde, (*E*,*E*)-2,4-hexadienal, hexanal, octanal, and heptanal were the volatiles with OAVs ≥ 1 in more than 65 tea plant cultivars. *Cis*-3-hexen-1-ol and (*E*)-4-hexen-1-ol (grassy and fruity aromas) as alcohols and 3-carene (citrus-like and woody aromas) as a hydrocarbon were the volatiles both with OAVs ≥ 1 in most of tea plant cultivars. Of the esters, only methyl salicylate (peppermint aroma) was a volatile with OAV ≥ 1 in some tea plant cultivars.

To distinguish the geographically characterized volatiles in different tea plant cultivars, differential metabolites were screened by using PCA (Figure 3b) and orthogonal projections to latent structures discriminant analysis (OPLA-DA) (Figure 3c) simultaneously. It was found that both models could achieve good separation. The PCA results show that the tea plant cultivars from Yunnan were separated from other regions, and most tea plant cultivars from Fujian were clustered with Zhejiang, Anhui, and Sichuan. In the permutation test of validation of the OPLS-DA model (Figure 3d), R^2^Y = 0.708 and Q^2^ = 0.0532, indicating a good model fit. Subsequently, the variable importance in the project (VIP) was calculated (Figure 3e), and the volatiles with both VIP > 1 and OAVs ≥ 1 were selected as potential difference volatiles for further identification. A total of five components were identified as characteristic volatiles in 70 tea plant cultivars, including methyl salicylate, 3-carene, *cis*-3-hexen-1-ol, (*E*)-4-hexen-1-ol, and 3-methylbutyraldehyde. Ultimately, the HCA of characteristic volatiles showed that tea plant cultivars from different regions could be clearly distinguished based on the above five volatiles (Figure 3f).

### 2.6. MGWAS Analysis and Candidate Gene Screening

**Figure 4:**
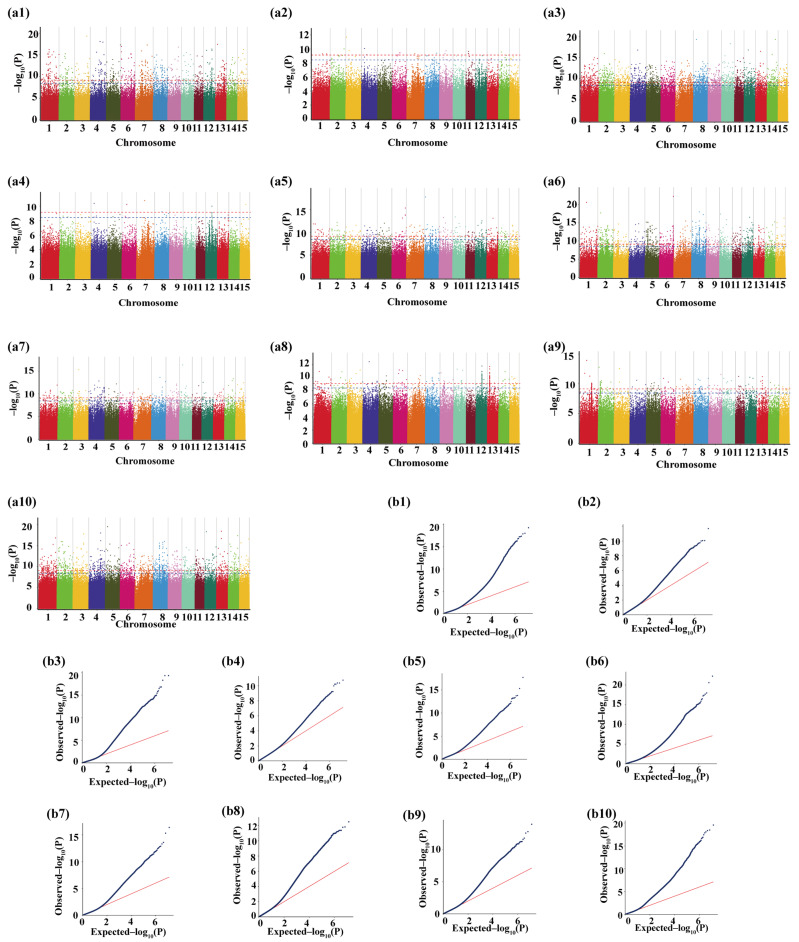
(**a1**–**a10**) Manhattan plots of α-farnesene association analysis (**a1**); *cis*-2-penten-1-ol association analysis (**a2**); 3-carene association analysis (**a3**); (Z)-linalool oxide association analysis (**a4**); (*E*,*E*)-2,4-hexadienal association analysis (**a5**); (*E*)-4-hexen-1-ol association analysis (**a6**); (*E*)-linalool oxide association analysis (**a7**); hexanal association analysis (**a8**); *cis*-3-hexen-1-ol association analysis (**a9**); methyl salicylate association analysis (**a10**); The blue and red dotted lines show significant associations between SNPs and metabolites value. (**b1**–**b10**) Quantile–Quantile (Q–Q) plots of the five traits in the same order with Manhattan plots showing the expected null distribution of *p*-value assuming no associations. The red line represents the predicted value and the blue dot represents the observed value, which can show the difference between the predicted value and the observed value.

It revealed that the decay distance of genome-wide LD for the whole population was from 0 to 50 kb, where the R^2^ dropped from 0.1 to 0.05 (Supplemental Figure S10a). Due to the faster rate of decay, we concluded that the correlation analysis was highly accurate. Ten characteristic volatiles (including α-farnesene, 3-carene, *cis*-2-penten-1-ol, (Z)-linalool oxide, (*E*,*E*)-2,4-hexadienal, (*E*)-4-hexen-1-ol, (*E*)-linalool oxide, hexanal, cis-3-hexen-1-ol, and methyl salicylate) from 70 tea cultivars were selected as phenotypic traits for subsequent mGWAS analysis. The skewness and kurtosis of the 10 traits were positive, and 3-carene had the highest kurtosis values and a high probability of data extremes (Table 1). Subsequently, association analysis was conducted between high-quality SNP loci and volatile phenotype data by using the MLM (K) model (Figure 4b1–b10). As a result (Figure 4a1–a10), α-farnesene and *cis*-2 pentan-1-ol were significantly associated with 963 and 56 markers and had the highest degree of association with Chr03_179317521 and Chr03_9258569, respectively. 3-carene (significantly associated with 2797 markers), (Z)-linalool oxide (significantly associated with 468 markers), (*E*,*E*)-2,4-hexadienal (significantly associated with 29 markers), (*E*)-4-hexen-1-ol (significantly associated with 1362 markers), and (*E*)-linalool oxide (significantly associated with 529 markers) had the highest degree of association with Chr14_122731139, Chr08_16489975, Chr07_118143306, Chr06_206575794, and Chr10_47770141, respectively. Hexanal was significantly associated with 298 markers and had the highest degree of association with Chr04_95153832, whereas *cis*-3-hexen-1-ol was significantly associated with 341 markers and had the highest degree of association with Chr01_95142047. Methyl salicylate was significantly associated with 1495 markers and had the highest degree of association with Chr05_33427085. Based on the SNP loci screened by mGWAS analysis and the reference genome, genes near 50 kb of significant loci on the reference genome were used as candidate genes. Subsequently, to screen the genes associated with aroma-related metabolic pathways, all candidate genes were subjected to GO and KEGG enrichment analysis, which showed that 38 aroma-related genes were found in α-farnesene, 154 for 3-carene, three for *cis*-2-penten-1-ol, 31 for (Z)-linalool oxide, one for (*E*,*E*)-2,4-hexadienal, 64 for (*E*)-4-hexen-1-ol, 41 for (*E*)-linalool oxide, 11 for hexanal, 16 for *cis*-3-hexen-1-ol, and 79 for methyl salicylate. The above 438 trait aroma-related genes were considered candidate genes.

### 2.7. Screening and Functional Analysis of Volatile-Associated Genes Subject to Selection Pressure

The analysis of selection pressure on tea plant cultivars in different regions showed that the nucleotide diversity in tea plant cultivars was high in Southern China, and the tea plant cultivars in Southern China were among the most distant from those in Fujian. Therefore, further analysis of the reasons for aroma differences between Fujian and Southern China during tea plant evolution was conducted. A total of 31 genes of all selected genes were found (*p* < 0.05); among them, five genes were significantly associated with α-farnesene, five for 3-carene, four for (*E*)-4-hexen-1-ol, eight for (*E*)-linalool oxide, and nine for methyl salicylate (Supplemental Figure S11). Furthermore, GO enrichment analysis of the above 31 genes revealed that these genes were mainly enriched in pathways related to metabolic and cells (Supplemental Figure S12). The results show that the ACT structural domain protein gene *ACR10*, LRR-like receptor serine/threonine protein kinase gene *At3g47570*, stress response protein NST1-like gene, pectin acetyl esterase gene *PAE8,* and 28S ribosomal protein genes were related to α-farnesene. Gag-Pol polyprotein gene, reactive oxygen species-related gene, O-fucosyltransferase gene *OFUT30*, WAT1 protein-related gene *At2g39510,* and pumilio domain-containing protein gene *APUM7* were related to 3-carene. AP-1 complex subunit gene *AP1M2*, solute carrier family 25 member gene *CIMG_07070*, receptor protein gene *RLP21*, and V-type proton ATPase gene were related to (*E*)-4-hexen-1-ol. Cytochrome C oxidase gene *COX2*, mitochondrial protein gene *AtMg00030*, ribosomal protein genes *RPS13* and *RPL5*, mitochondrial complex III subunit gene *MT-CYB*, WAT1 protein-related gene *At2g39510*, and pumilio domain-containing protein gene *APUM7* were related to (*E*)-linalool oxide. The genes related to methyl salicylate included chaperone protein genes, receptor-like protein 14 genes *RLP21* and *RLP14*, F-box/kelch-repeat protein gene *At1g60070*, nuclear pore complex protein gene *NUP98A*, E3 ubiquitin-protein ligase gene *RFI2*, and some unknown function genes.

## 3. Discussion

As a cash crop in China, the research of tea plant *cultivars* is the key to improve the tea quality [28]. Lou et al. studied the diversity in biochemical components of 76 tea varieties in Lishui, proving that tea plant *cultivars* have great selection potential and rich diversity [29]. In this study, we found that genes related to the quality and photosynthesis of tea plants were all subjected to selection during the evolutionary process, which is of great significance for breeding new tea plant varieties with high quality. Aroma plays a vital role in the quality of tea, and in addition to the tea manufacturing process, tea cultivars are the basis for the formation of tea aroma [8]. The differences in aroma of different tea plant varieties have been revealed and the candidate genes associated with volatiles were identified in this study, which will provide a reference for the establishment of highly aromatic core tea *plant cultivars* in Fujian.

### 3.1. Extensive Exchanges between the Tea Plant Cultivars in Fujian and Other Regions

Genetic diversity analysis has always been of great significance for the evaluation and utilization of crop germplasm, as well as for the study of origin and evolution [30]. Analysis of population structure demonstrated that tea cultivars in thirteen provinces were clustered into three groups (Figure 1b). However, most of the tea plant cultivars among the subgroups were genetically infiltrated, except for those in Northern Fujian, Yunnan, and Guangdong, indicating frequent genetic exchange among tea plants, which is consistent with the results of previous studies [2,26,31]. The Anxi in Southern Fujian and Fuding in Eastern Fujian tea populations are highly divergent, while the Wuyishan and Zhenghe in Northern Fujian have a high frequency of gene exchange with the Anxi in Southern Fujian and Fuding and Fuan in Eastern Fujian tea populations, which was confirmed by Nie et al. [32] These results will provide a reference for the study of molecular breeding of tea plant in Fujian Province. By analyzing the genetic diversity in oolong tea plant cultivars, it was found that Taiwan and Fujian oolong teas clustered into one taxon and there was a genotypic intersection between Southern Fujian and Northern Fujian tea plant cultivars [33]. Wuyi Mountain in Fujian province is an important mountain range in Eastern China, spanning Fujian, Guangdong, Jiangxi, and Zhejiang from north to south, which gradually changed from ocean to land during the Cretaceous Period of the Mesozoic Era, and then rose during the Ice Age at the end of the Quaternary Era, resulting in the creation of folded and fractured valleys between the mountains [34]. The geological age of Wuyi Mountain, the rocky nature of the soil and the presence of wild tea are a series of characteristics that suggest that the tea plant of Wuyi Mountain evolved from a homologous “isolated distribution” into the “Wuyi taxon” [35]. “Wuyi Caicha” occupies an important position in the taxonomy of tea plants, and TLH, SJG, BJG, and BTY were bred from it [36]. Therefore, we speculated that some tea plants in Fujian may have developed and evolved from northern Fujian due to geological changes [35,37].

### 3.2. Genes Related to Quality and Photosynthesis of Tea Plants May Be Selected during Evolutionary Processes

Tea quality has always been the focus of tea research. Accurate targeting of tea quality-related genes during tea plant evolution is significant for the cultivation of new high-quality tea plant varieties [38]. In this study, the annotation of selected genes by selection pressure analysis revealed that the gene *ACR10* was selected in all four populations (Supplemental Figure S6). The gene *ACR10* is involved in encoding ACR proteins, and all ACR proteins with multicopy ACT domains bind amino acids. For example, 3-phosphoglycerate dehydrogenase, which catalyzes the first step of serine synthesis, can bind serine; and branch acid translocase, which catalyzes the first two steps of phenylalanine synthesis, can bind phenylalanine. In other words, the terminal product amino acid can be bound to the ACT domain of its synthase, and the activity of the synthase can be regulated according to the concentration of the product amino acid, thus regulating the metabolism of the amino acid [39,40,41]. At present, 26 amino acids have been identified in tea leaves, which play an important role in the function and quality of tea leaves and their growth [42,43]. It was found that there were significant differences in amino acid contents among different groups of tea plant cultivars by analyzing the diversity in biochemical components of tea plant cultivars [44]. This may be the reason why tea plant cultivars from the Fujian region differ from those of other regions in terms of tea quality [45]. The selected differential genes in tea plant cultivars from ECF and CCF are mainly associated with enzyme activities that regulate plant growth and development, among which *SAMDC*-encoding S-adenosylmethionine decarboxylase was involved in caffeine biosynthesis. Caffeine plays an important role in plant defense against biotic stresses as a secondary metabolite of tea leaves, and its content, together with tea polyphenols, determines the suitability of *Camellia sinensis* for tea production and tea flavor [46,47]. Similar results were found in the study by Yang et al., suggesting that genes associated with tea leaf quality in tea plants were selected during long-term evolution [24].

In mainland China, the highly suitable area for tea planting covers about 16.83% of the continental area [48]. Studies have shown that climate change has important effects on plant growth and geographic distribution, resulting in large differences in genetic diversity, geographic distribution, community composition, and function among different tea plants [49]. Most of the selected genes were found to be involved in regulating photosynthesis and respiration in plants in this study (Supplemental Figure S6). As an important climatic condition, light is inseparable from the growth and development of plants. By analyzing the leaves of tea plants, from Guizhou, Sichuan, Hunan, Fujian, Zhejiang, and Guangdong provinces, which were planted in the same region, it was found that the differences between photosynthetic pigments and net photosynthetic rate were due to the genetic characteristics of the tea varieties [50]. Additionally, the geographic distribution of the tea plants was affected by the climate environment with regularity [51]. There was diversity in photosynthesis and physiological activity among different genetic populations of tea plants, and rich genetic variation in photosynthetic characteristics during adaptation to new ecological environments [52]. The climate in China is complex and diverse, and plant photosynthesis is susceptible to environmental stress; thus, variation in photosynthesis-related genes under selection pressure in different regions has led to differentiation among populations of tea plants [53]. Hence, we hypothesized that photosynthesis plays an important role in the evolution of tea plant propagation in terms of its adaptive capacity, resulting in genetic variation among populations of tea plants distributed in different regions with different genetic characteristics.

### 3.3. Selection-Pressured Volatile-Associated Genes May Be Markers for Screening Highly Aromatic Tea Plant Cultivars

Although the climate, soil conditions, and mountainous topography of both Fujian and Southern China are similar [54,55], there is a significant genetic gap between the two tea cultivars. It has been shown that there exists a high level of metabolite diversity in different tea populations and varieties, and that genetic background has a stronger effect on metabolite contents than environmental factors [56]. The 70 tea plant cultivars selected for this study were all planted in the same cultivation system; thus, it was inferred that the differences between varieties caused the differences in volatile fractions between tea cultivars in Fujian and South China. A total of 31 genes were screened by the association analysis of selection pressure and volatiles (Supplemental Figure S11), and some of them were associated with two volatiles. In this study, 3-carene, a volatile with a citrus-like aroma, was the main contributor to the volatiles from different tea plant cultivars. (Supplemental Table S6). Among the five genes significantly related to 3-carene, the Gag-pol polymer gene was involved in resistance regulation during plant growth [57,58]; The pumilio proteins may be involved in a wide range of post-transcriptional regulatory events, which are important for the rapid response of plants to changes in environmental conditions and throughout the developmental process [59]. Linalool and its oxides, α-farnesene, etc., as important aromatic compounds in the leaves of the highly aromatic tea plant variety HD were found by Wang et al. [8]. The *ACR10* gene is involved in encoding the acr protein, which binds to phenylalanine [60,61]. In addition, aromatic cyclic substances in tea are synthesized from phenylalanine via the shikimic acid pathway and are the main components of the fruity aroma of tea leaves. Cytochrome C oxidase is mainly involved in respiration, which lays the foundation for the formation of aroma and flavor by facilitating the transformation of the intrinsic qualities of tea leaves [62]. As described, all these volatile-associated genes subject to selection pressure were involved in regulating plant growth processes and associated with highly aromatic volatiles. Thus, these genes may have been involved in the formation of aroma during the evolution of tea plants and were key genes responsible for the formation of aroma differences between tea plant cultivars in Fujian and Southern China, which can be used as markers for screening highly aromatic tea plant cultivars.

## 4. Materials and Methods

### 4.1. Sample Collection

To investigate the origin of tea plant in Fujian Province and to explore the genes related to the flavor quality of tea plant, a total of 70 tea plant cultivars (Supplemental Table S1), which are representative species from different tea zones, including Western, Central, Southern, and EC-F were selected. All samples were collected from the tea germplasm nursery of the Tea Research Institute of Fujian Academy of Agricultural Sciences (119°34′ E, 27°13′ N) and the tea germplasm resource nursery with excellent characteristics in Fujian and Taiwan of Wuyixing Tea Industry Co., Ltd., Fujian, China (118°0′ E, 27°43′ N). In the spring of April 2021, a bud with two leaves of tea plants with good growth were taken as materials, and the samples were stored in the refrigerator at −80 °C for standby after solid sampling with liquid nitrogen.

### 4.2. DNA Extraction, Library Construction, Sequencing, and Data Processing

Genomic DNA was extracted from the 70 tea plants by using the CTAB method [63]. DNA purity and concentration were tested by using Qubit (Thermo Fisher Scientific, Waltham, MA, USA) and Nanodrop (Thermo Fisher Scientific, Waltham, MA, USA). Based on the published genomic data for comparison, the genome with the highest comparison rate was selected as the reference genome. As far as possible to test the efficiency of enzymatic digestion and analyze all markers, the number and coverage of enzymatic segments were counted by electron microdissection prediction of the reference genome using the restriction endonuclease EcoR I (G, AATTC). After that, a series of operations were conducted, including fragment end reparation, paired-end adapter ligation, polymerase chain reaction (PCR), and library construction [64]. Ultimately, sequencing was carried out using the PE 150 strategy on a Novaseq 6000 sequencer (Illumina, San Diego, CA, USA) at Kidio Biotechnology Co., Ltd. in Guangzhou, China. The sequencing original data from the Illumina platform were screened through FASTP software (version 0.18.0). Reads with unknown nucleotides (N) ≥ 10%, Phred quality score ≤ 20 with more than 50% base content and those containing connectors were excluded [65]. Subsequently, the filtered reads were compared to the reference genome using the software BWA (version 0.7.12). Then, the alignment results were tagged and population SNPs were detected by using Picard (version 1.129) and GATK (version 4.0), respectively.

### 4.3. Population Structure, Principal Component, and Phylogenetic Tree Construction Analysis

Based on the SNPs obtained, the population structure of 70 tea plants was conducted by using Admixture (version 1.3.0), and the tested K was set from 1 to 9 [66]. According to the cross-validation method, the best genetic cluster K was determined and then the results graph was plotted with R language. PCA was conducted by using GCTA (version 1.92.2) based on the degree of SNP variation among tea plant samples, and PCA scatter plots were drawn using the R language. A phylogenetic tree was conducted by using MEGA (version 4.0) according to the maximum likelihood algorithm with 1000 bootstrap replicates.

### 4.4. Select Pressure Analysis

To identify potential selective regions, intra-population nucleotide diversity (π), neutrality test (Tajima’s D), genetic differentiation index (Fst), and diversity difference ratio (π ratio) were analyzed using PopGenome (version 2.7.5) with a 100 kb sliding window and a step size of 10 kb [67,68]. Regions subject to selection tend to be chromosomal regions with low genetic diversity within populations, as well as regions with high rates of genetic differentiation between populations. This combination is based on the results of Fst, π, and π ratio, each of which is screened for significant regions by top 1% and top 5%. The regions, with decreasing heterozygosity (−log10π ≥ threshold), significant differences between populations (Fst ≥ threshold), and diversity differences between populations (π ratio ≥ threshold) were obtained, and the intersection of these regions was located as potential selected regions, and the genes in these selected regions were identified. Ultimately, Venn diagram mapping, as well as GO and KEGG annotation of the selected genes, were conducted based on the selected genes, and subsequent analysis was conducted for variants such as SNPs and InDel in the coding regions of the candidate genes.

### 4.5. Analysis of Volatiles

The test tea samples were ground into tea powder of 40 mesh sizes after freeze-drying for 48. A total of 0.5 g of the powder was transferred to a 20 mL headspace vial and infused with 3 mL saturated NaCl solution. Subsequently, an internal standard of 5 μL 4-tert-butylcyclohexanol (30 mg∙L^−1^) was added into the vial, and then the vial was sealed immediately.

The method of volatile detection was adjusted by referring to the method of Wei Wang et al. [69]. GC conditions: 50 °C held for 5 min, then increased to 125 °C at a rate of 3 °C/min, then held for 2 min; and to 180 °C at a rate of 5 °C/min, then held for 3 min; and last to 230 °C at a rate of 15 °C/min, then held for 5 min. The carrier gas was helium (percentage purity > 99.999%) at a constant flow velocity of 1 mL/min. MC conditions: Electron ionization source; electron energy, 70 eV; ion source temperature, 230 °C; mass spectrometry transmission line temperature, 250 °C; mass scan range 45–500 AMU.

The volatiles were identified tentatively by comparing their mass spectra from the NIST11 standard reference database and further positively identified with retention indices, retention times, and the data available in the published literature. The content of each substance was calculated using 4-tert-butylcyclohexanol as the internal standard [70]. Odor activity value (OAV) is commonly used to evaluate the contributions of each volatile to the aroma of a sample. Numerically, OAV is equal to the ratio of the compound concentration (Ci) to the odor threshold (OTi) in water, and compounds with OAV > 1 are generally considered to greatly contribute to the aroma characteristics [70].

### 4.6. Genome-Wide Association Analysis

To identify SNP loci and candidate genes associated with tea plant aroma traits, variant quality control was conducted based on the above SNP loci. The raw marker loci were filtered by self-written Perl scripts combined with Plink software (version 2.0). Loci with non-double allelic loci, minor allele frequency < 0.05, deletion rate > 0.5, and heterozygosity rate > 0.8 were filtered. Phylogenetic tree construction, PCA, LD decay analysis, population structure analysis, and genetic relationship analysis were conducted based on filtered loci (Supplemental Figure S10). And based on the results of volatile detection, descriptive statistical analysis of the volatile content of different species was conducted using R language.

The above SNP data were used for genome-wide association analysis with the phenotypic trait data of volatiles of 70 tea plant cultivars. The mGWAS model was calculated by Gemma software (version 0.94.8), where the top five PCs of the PCA calculation results were used as the Q matrix of the corresponding model, and the genetic relationship matrix was used as the K matrix of the corresponding model. A total of four models, a general linear model (GLM), a generalized linear model with correction for PCA results (GLM (Q)), a mixed linear model with correction for genetic relationship results (MLM (K)), and a mixed linear model with correction for PCA and genetic relationship results (MLM (QK)) were used for pre-analysis in this study. The final analysis model was determined according to the deviation between the actual value and the expected value of the Q-Q plot.

## 5. Conclusions

In this study, the origin and evolution of tea plants in Fujian province were revealed by analyzing the genetic diversity and genetic relationship in tea plants. By analyzing the aroma components of tea plants, differences in the aroma of tea plant cultivars were identified and candidate genes associated with volatiles were mined. Based on selection pressure and association analysis, key genes causing differences in tea plant aroma in different regions were screened. All these aroma-related genes among different tea plant cultivars may be developed into a genetic marker to be used in the identification process of breeding.

## Figures and Tables

**Table 1:** Characteristics of volatiles in fresh tea leaves.

Trait	Mean	Standard Deviation	Standard Error	Median	Min	Max	Range	Skew	Kyrtosis
α-farnesene	965.16	1710.24	204.41	300.42	5.5	10,144.3	10,138.79	3.45	13.75
3-Carene	2804.67	4639.92	554.58	1260.3	29.59	33,451.74	33,422.15	4.38	24.73
*Cis*-2-pentenol-1-ol	315.86	363.03	43.39	132.32	12.09	1681.63	1669.54	1.69	2.72
(Z)-linalool oxide	258.42	362.56	43.33	129.3	8.39	2141.99	2133.6	2.82	9.68
(*E*,*E*)-2,4-hexadienal	168.19	183.01	22.03	89.2	6.43	809.91	803.48	1.56	1.91
(*E*)-4-hexen-ol	879.54	1552.29	185.53	353.97	7.99	8209.08	8201.09	3.04	9.39
(*E*)-linalool oxide	615.06	920.17	109.98	272.16	19.98	6074.06	6054.08	3.06	15.51
Hexanal	184.14	244.71	29.25	73.66	3.89	1314.8	1337.91	2.23	5.88
*Cis*-3-hexen-1-ol	768.71	1054.45	126.03	350.32	1.61	6203.63	6202.02	2.57	8.55
Methyl salicylate	149.29	247.97	29.64	52.39	6.24	1585.38	1579.14	3.63	15.81

## Data Availability

All of the raw sequencing data were submitted to the National Genomics Data Center (NGDC) under project number PRJCA018106.

## Supplementary Materials

The following supporting information can be downloaded at: https://www.mdpi.com/article/10.3390/plants13010100/s1, Figure S1: (a) Statistics on the percentage of SNP types. (b) Insertion deletion variant loci statistics; Figure S2: Nucleotide diversity, neutrality test, and genetic index relationships of tea plants in various regions. FJ: Fujian; EC: Eastern China excluding Fujian (EC-F); WC: Western China; CC: Central China; SC: Southern China; Figure S3: (a–e) Nucleotide diversity in tea plants in different regions. (a) Fujian; (b) EC-F; (c) Southern China; (d) Central China; (e) Western China; (f–i) Genetic differentiation index of tea plants in different regions. (f) EC-F and Fujian (ECF); (g) Southern China and Fujian (SCF); (h) Central China and Fujian (CCF); (i) Western China and Fujian (WCF); Figure S4: Top 5% selected regions of the genome. (a1–a3) are ECF; (b1–b3) are SCF; (c1–c3) are CCF; (d1–d3) are WCF; Figure S5: A number of genes identified in the top 5% of selected genome regions. (a) ECF; (b) SCF; (c) CCF; (d) WCF; Figure S6: Top 20 GO annotations of selected genes in each region. (a) ECF; (b) SCF; (c) CCF; (d) WCF; Figure S7: Top 20 KEGG annotations of selected genes in each region. (a) ECF; (b) SCF; (c) CCF; (d) WCF; Figure S8: The proportion of volatile components in fresh tea leaves by region. (a) Western China; (b) Central China; (c) Southern China; (d) EC-F; (e) Fujian; Figure S9: The content of volatile components in fresh tea leaves; Figure S10: (a) Linkage disequilibrium; (b) Phylogenetic tree based on filtered SNPs; (c) Principal component analysis (PCA) based on filtered SNPs; Figure S11: Selective pressure volatile-associated genes; Figure S12: GO annotation of genes that were significantly associated with selected genes and traits. (a) α-farnesene; (b) 3-carene; (c) (*E*)-4-hexen-1-ol; (d) (*E*)-linalool oxide; (e) methyl salicylate. Table S1: Geographical origin distribution of 70 tea plant cultivars; Table S2: Sequencing data quality summary; Table S3: Filter before and after base information; Table S4: Number of SNPs on tea plant chromosomes; Table S5: Volatile content of fresh tea leaves; Table S6: OAV value of tea plant fresh leaf volatiles. Note: VC means volatile component.
