# Supplementary material for: Analysis of the Genetic Diversity in Tea Plant Germplasm in Fujian Province Based on Restriction Site-Associated DNA Sequencing
Source: Plants (Basel). 2023 Dec 28;13(1):100. doi: 10.3390/plants13010100 (PMC10780744; doi:10.3390/plants13010100)
Supplement: Supplementary file 1 [file plants-13-00100-s001.zip › Supplemental Figures.pdf]

Supplemental Figures

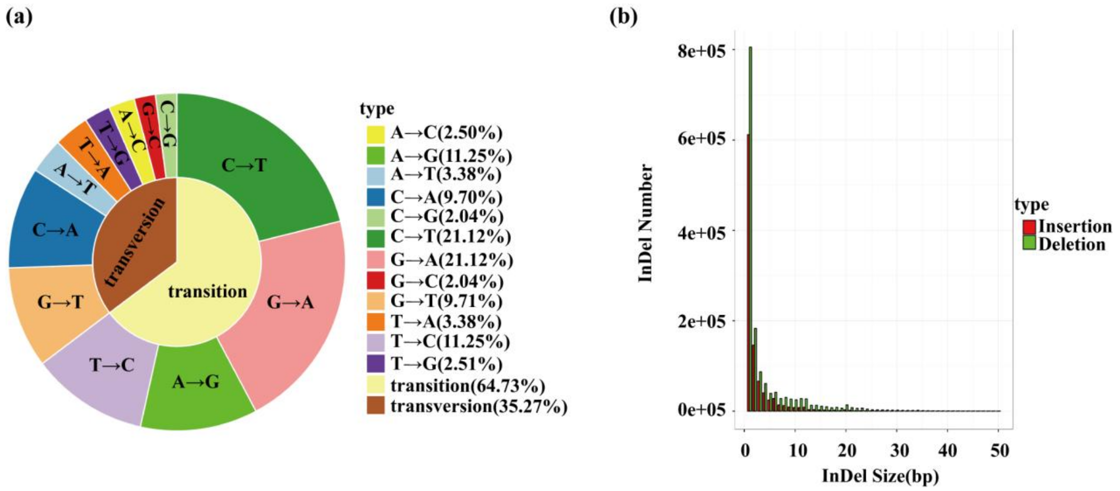

Supplemental Figure S1. (a) Statistics on the percentage of SNP types. (b) Insertion deletion variant loci statistics.

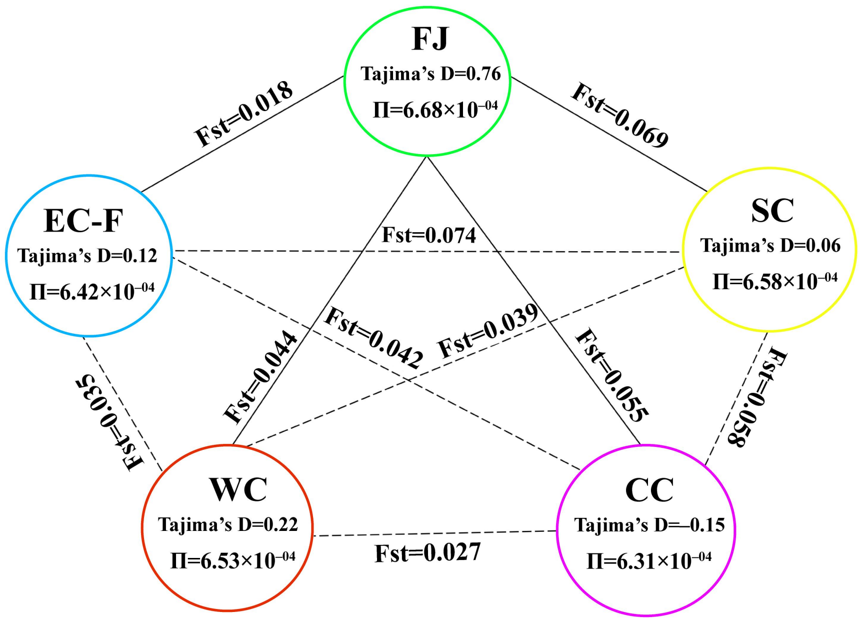

Supplemental Figure S2. Nucleotide diversity, neutrality test and genetic index relationships of tea plants in various regions. FJ: Fujian; EC-F: Eastern China excluding Fujian; WC: Western China; CC: Central China; SC: Southern China

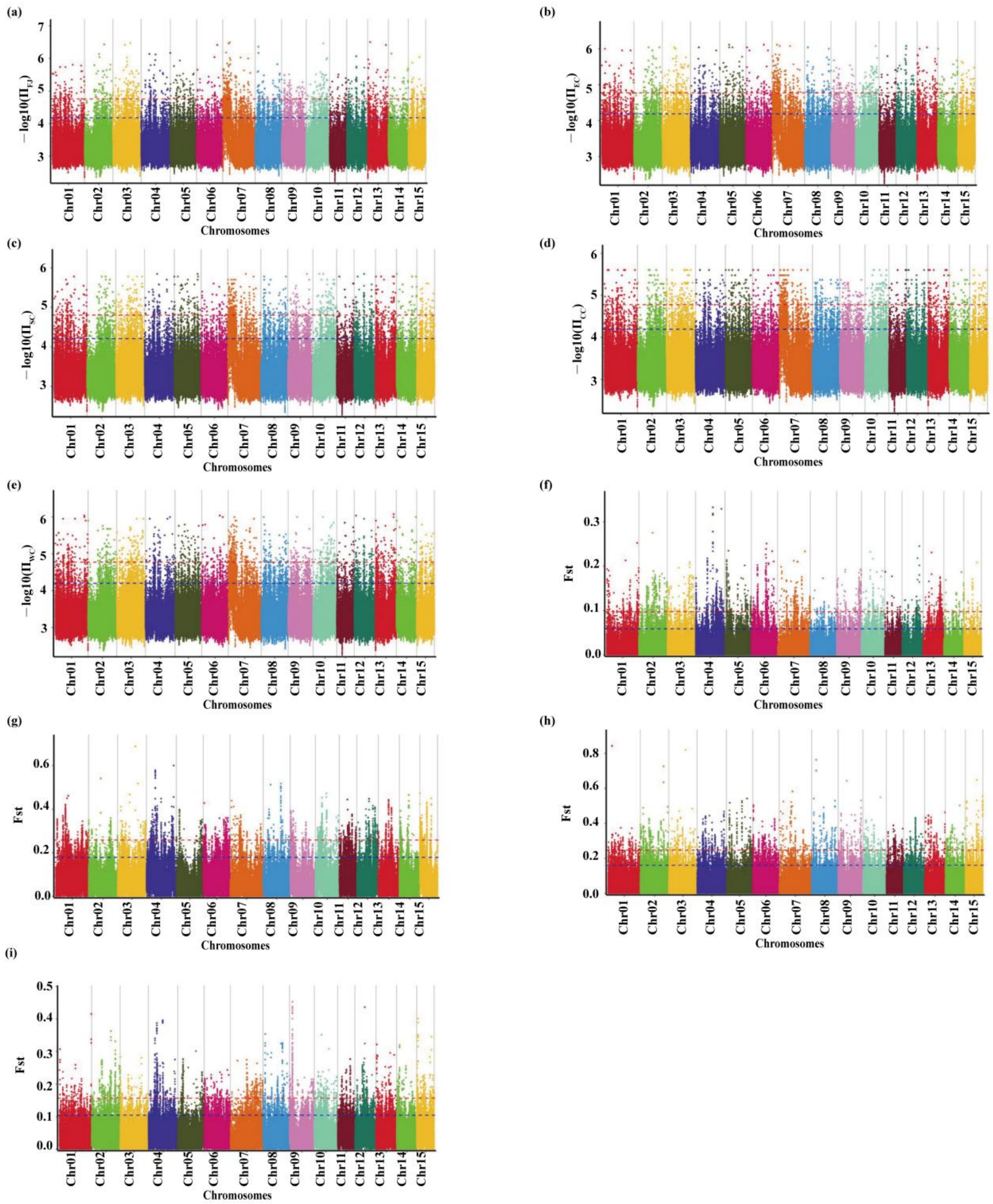

Supplemental Figure S3. (a-e) Nucleotide diversity of tea plants in different regions. (a) Fujian; (b) Eastern China excluding Fujian (EC-F); (c) Southern China (d) Central China; (e) Western China; (f-i) Genetic differentiation index of tea plants in different regions. (f) EC-F and Fujian (ECF); (g) Southern China and Fujian (SCF); (h) Central China and Fujian (CCF); (i) Western China and Fujian (WCF);

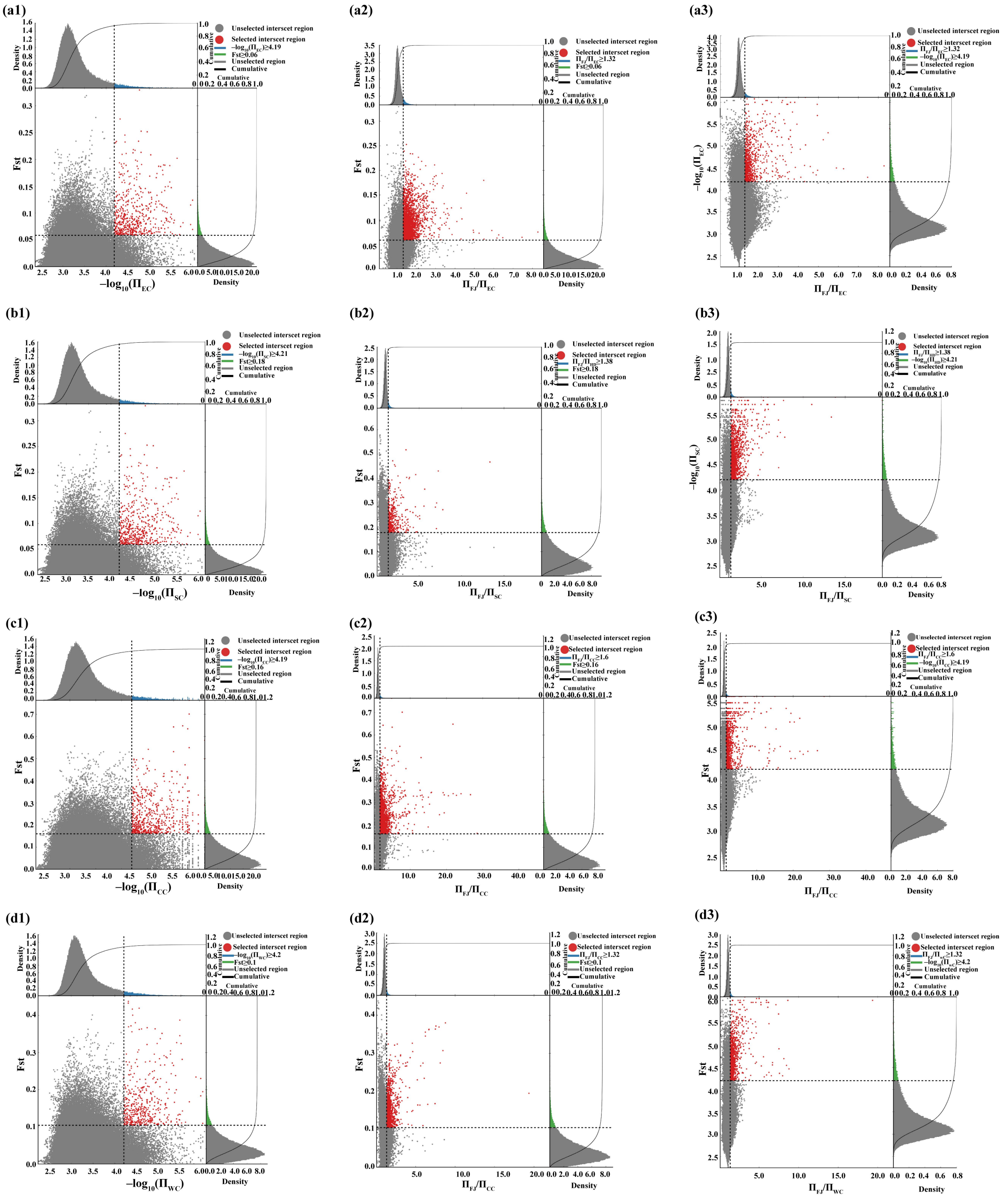

Supplemental Figure S4. Top 5% selected regions of the genome. (a1), (a2), and (a3) are ECF; (b1), (b2), and (b3) are SCF; (c1), (c2), and (c3) are CCF; (d1), (d2), and (d3) are WCF.

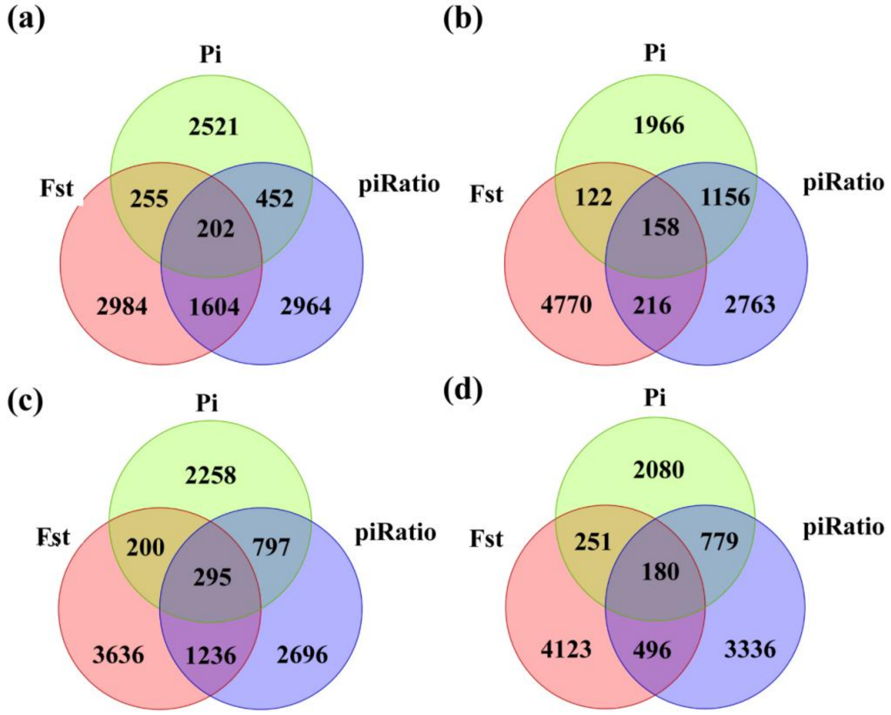

Supplemental Figure S5. A number of genes identified in the top 5% of selected genome regions. (a) ECF; (b) SCF; (c) CCF; (d) WCF.

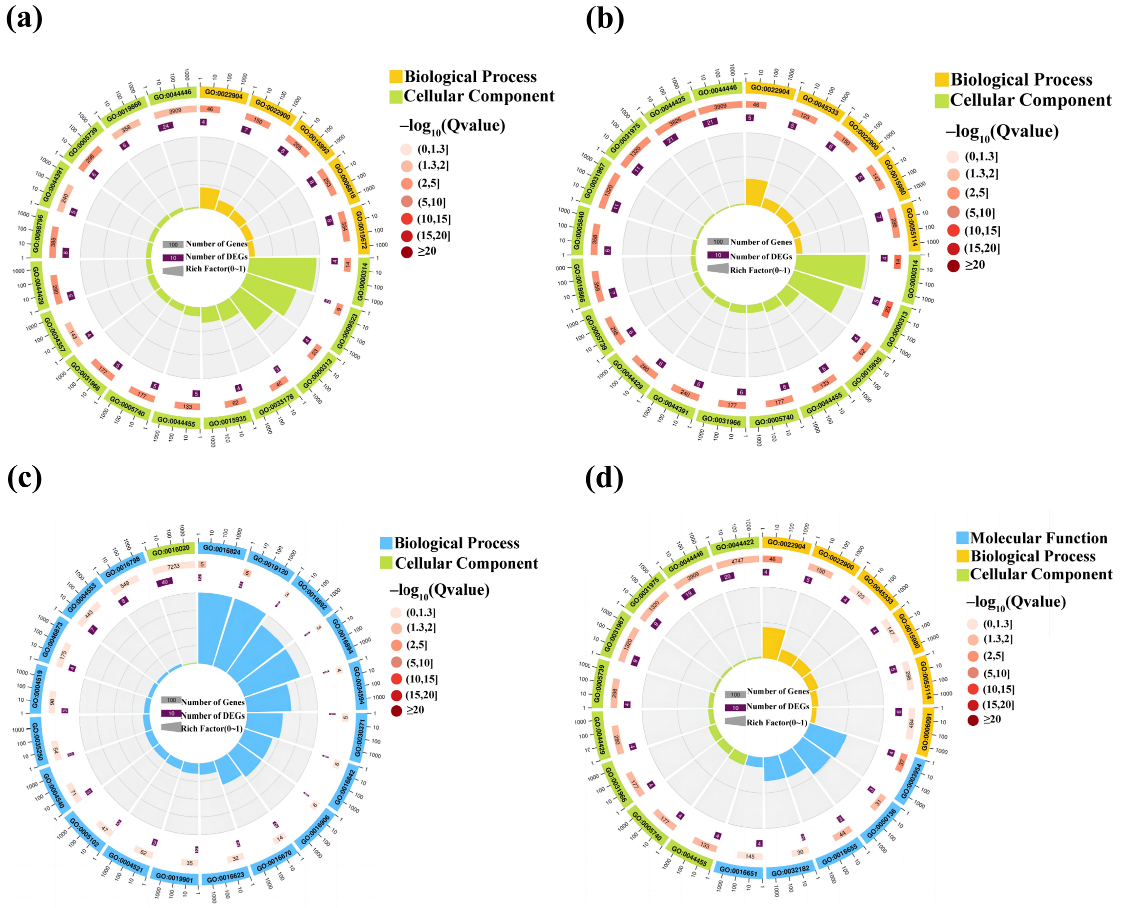

Supplemental Figure S6. Top 20 GO annotations of selected genes in each region. (a) ECF; (b)

SCF; (c) CCF; (d) WCF.

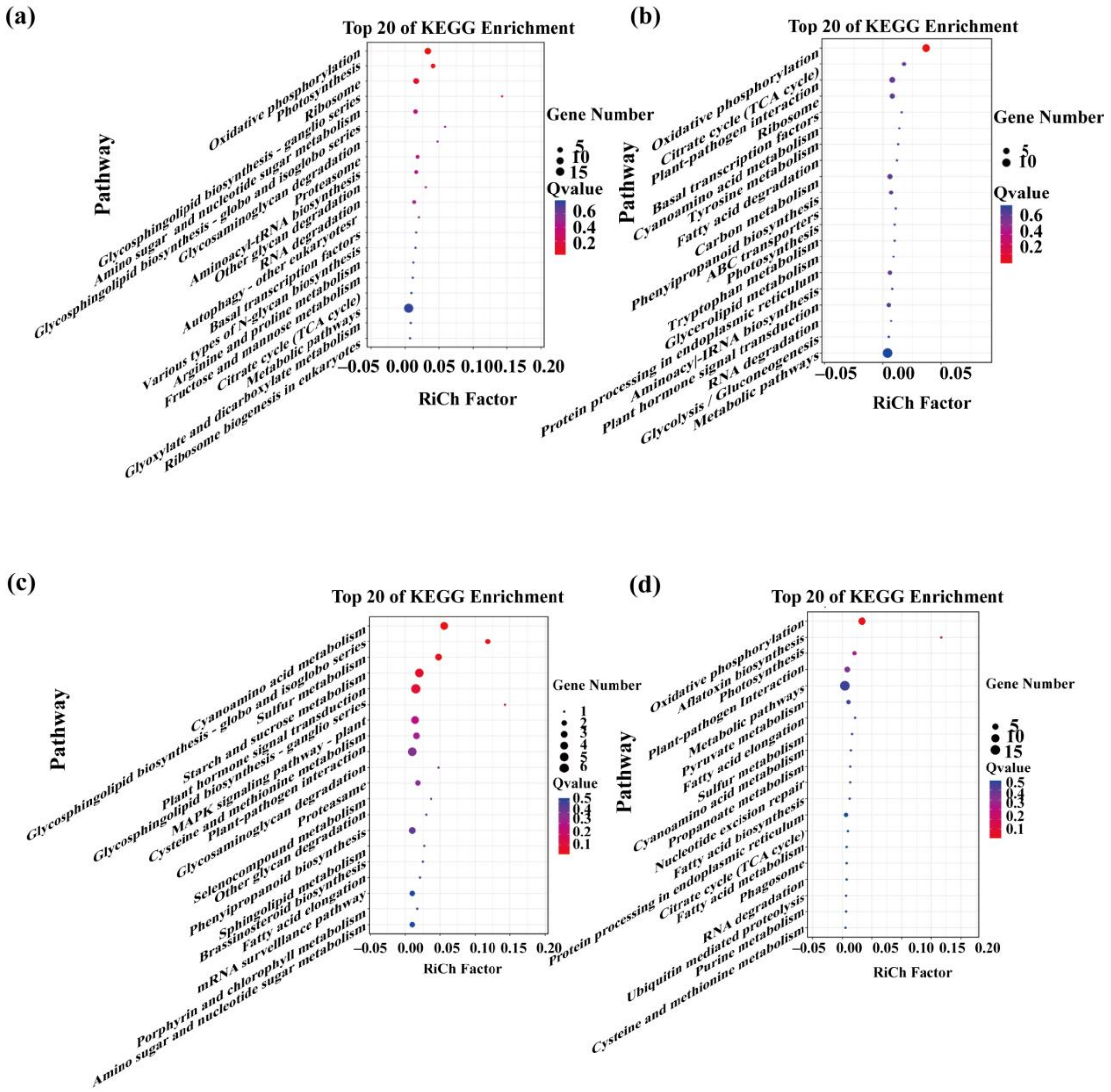

Supplemental Figure S7. Top 20 KEGG annotations of selected genes in each region. (a) ECF; (b) SCF; (c) CCF; (d) WCF.

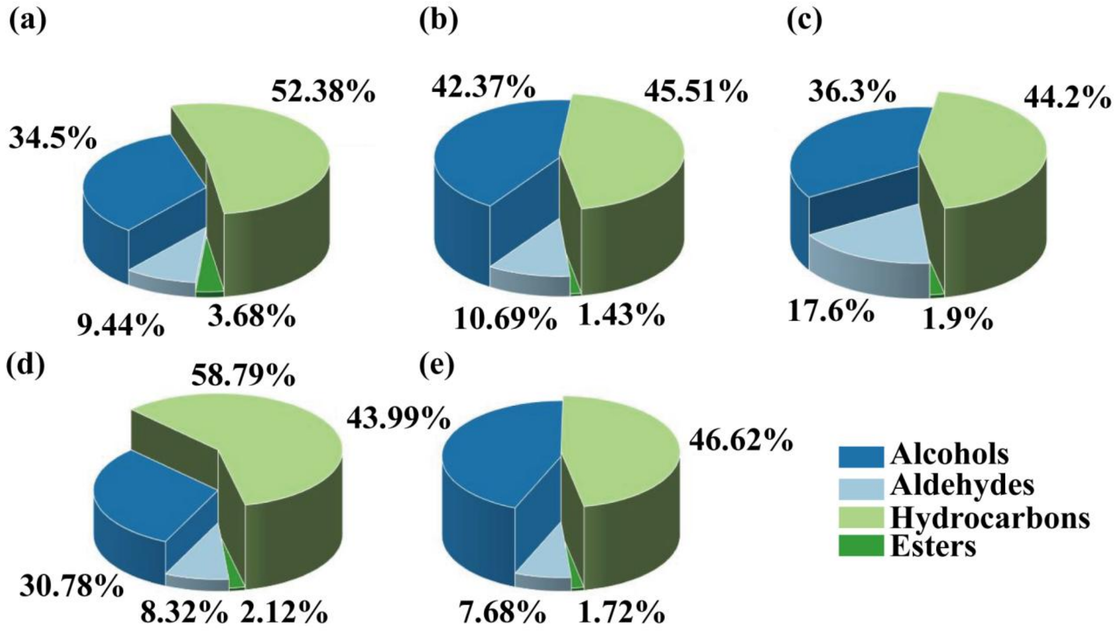

Supplemental Figure S8. The proportion of volatile components in fresh tea leaves by region. (a) Western China; (b) Central China; (c) Southern China; (d) EC-F; (e) Fujian.

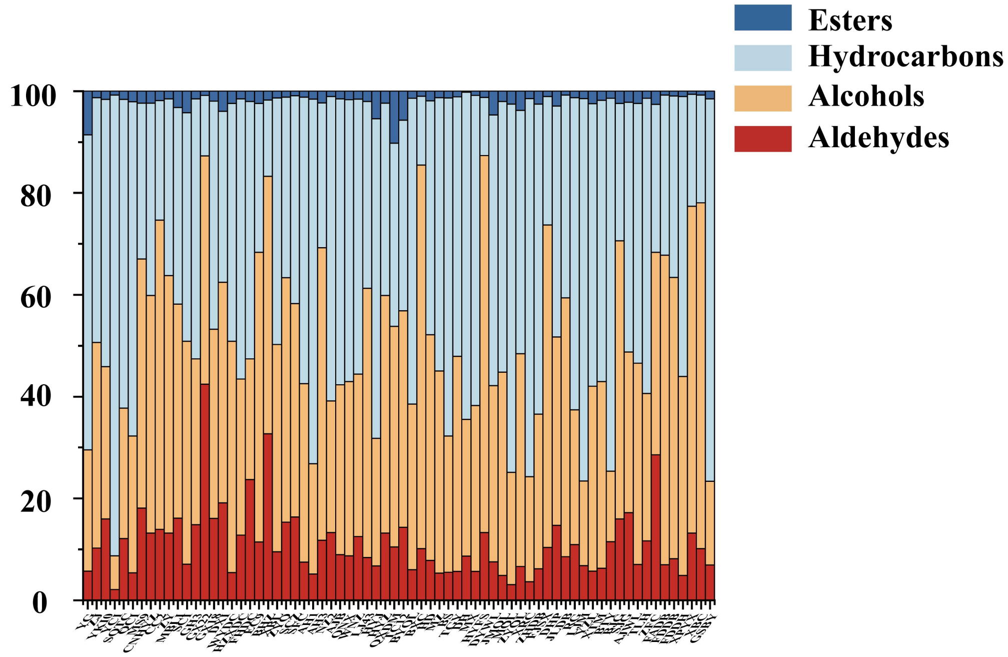

Supplemental Figure S9. The content of volatile components in fresh tea leaves.

(a)

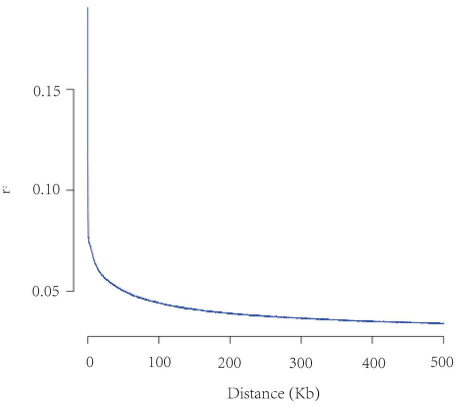

(b)

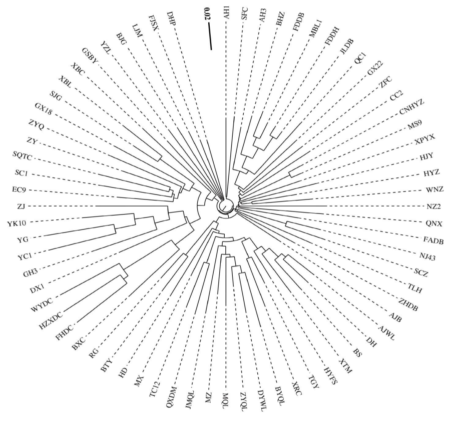

(c)

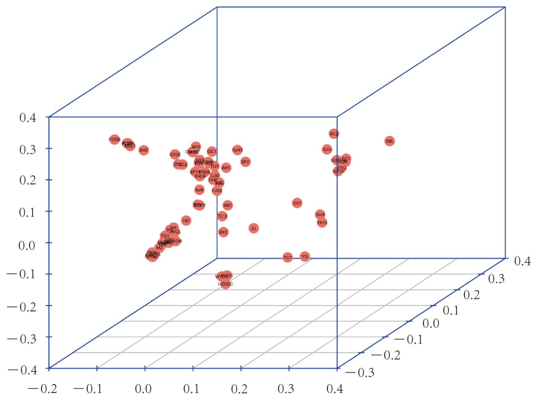

Supplemental Figure S10. (a) Linkage disequilibrium; (b) Phylogenetic tree based on filtered SNPs; (c) Principal component analysis (PCA) based on filtered SNPs.

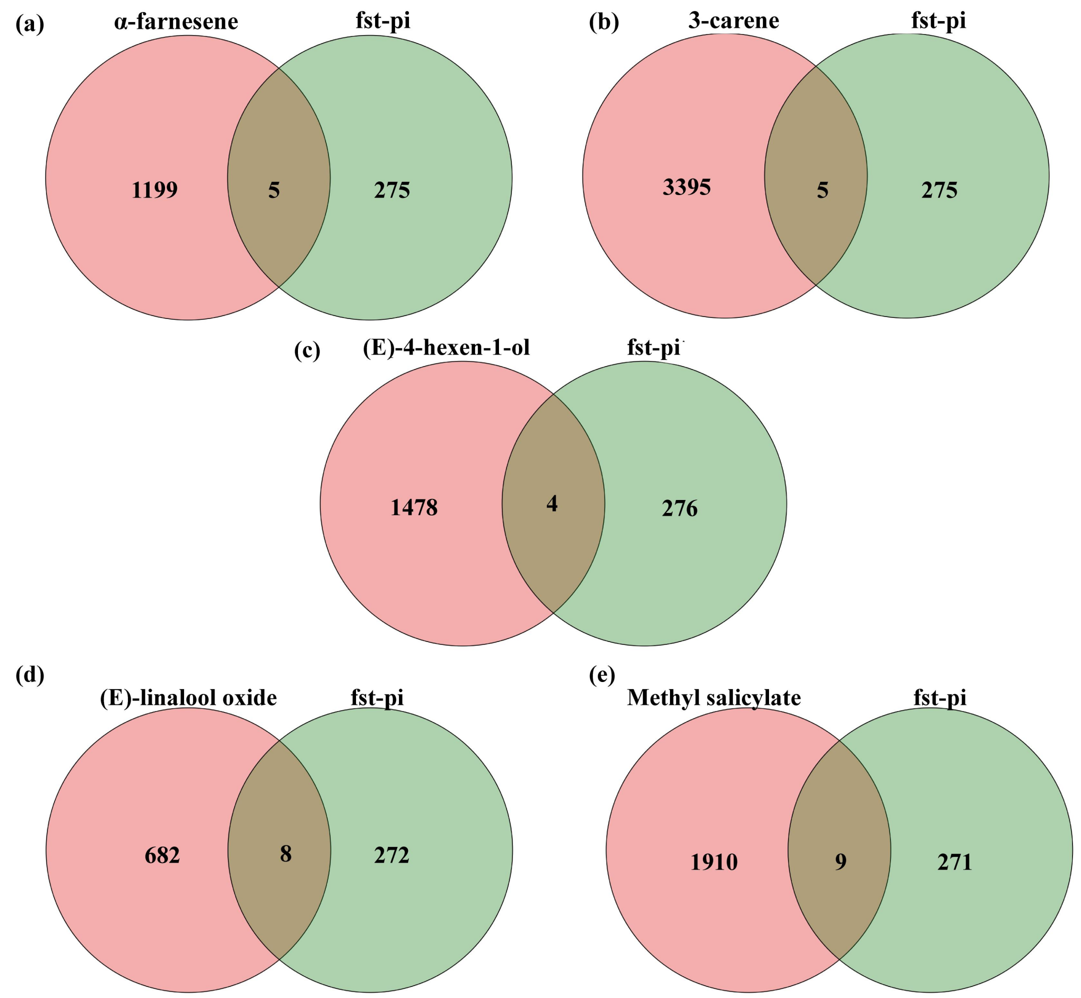

Supplemental Figure S11. Selective pressure volatile-associated genes.

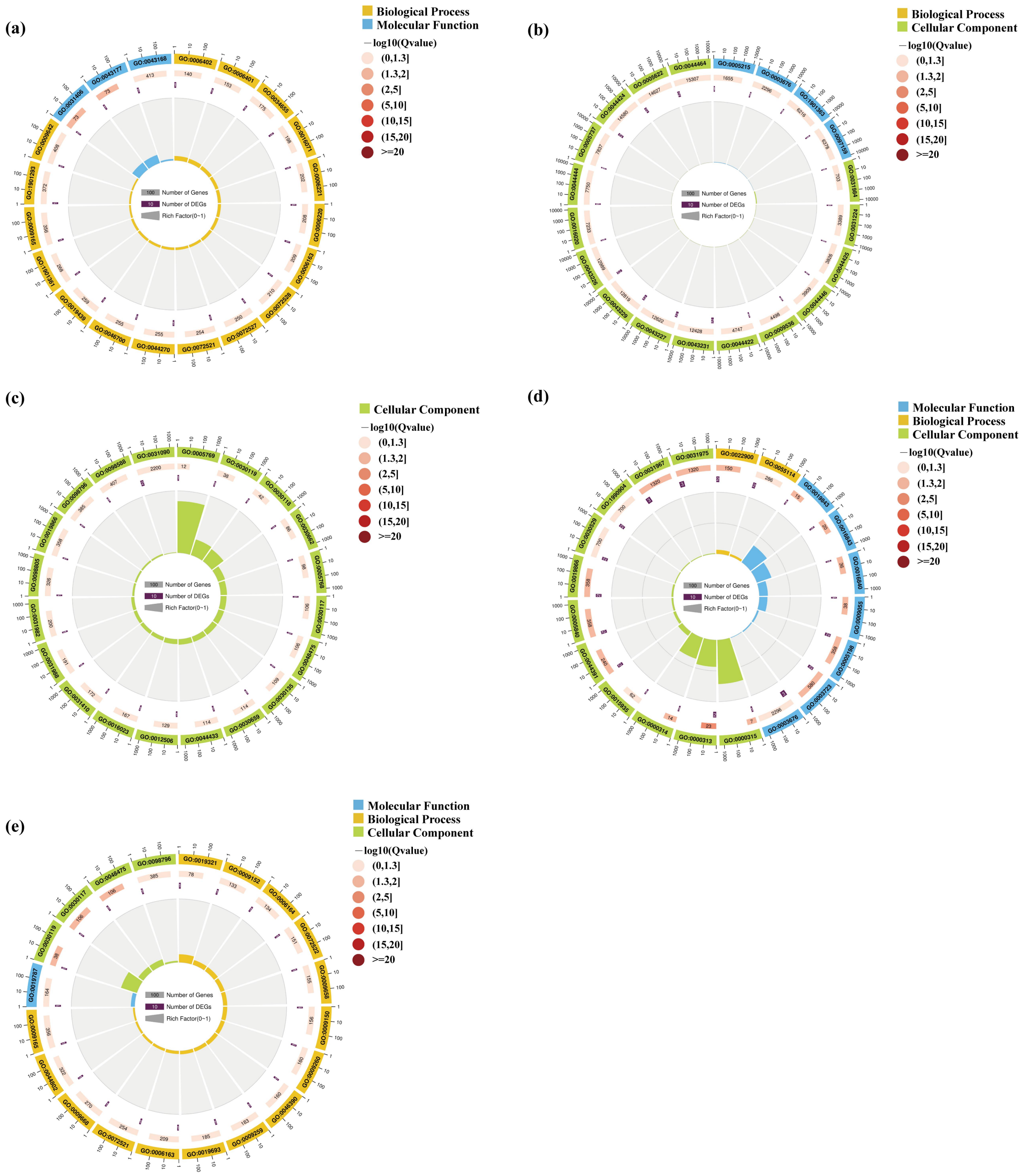

Supplemental Figure S12. GO annotation of genes that were significantly associated with selected genes and traits. (a) α-farnesene; (b) 3-carene; (c) (E)-4-hexen-1-ol; (d) (E)-linalool oxide; (e) methyl salicylate.
